# Use of natural ingredients in Japanese quail diet and their effect on carcass and meat quality — A review

**DOI:** 10.5713/ajas.18.0800

**Published:** 2019-02-09

**Authors:** Rey David Vargas-Sánchez, Félix Joel Ibarra-Arias, Brisa del Mar Torres-Martínez, Armida Sánchez-Escalante, Gastón Ramón Torrescano-Urrutia

**Affiliations:** 1 Meat and Meat Products Research Laboratory, Department of Technology of Animal Origin Food, Research Center for Food and Development, A.C. Hermosillo Sonora, ZIP 83304, Mexico; 2 Alta Tecnología Industrial para la Salud Animal, S.A. de C.V. Guadalajara Jalisco, ZIP 44430, Mexico

**Keywords:** Japanese Quail, Animal Diet, Natural Ingredients, Carcass Quality, Meat Quality

## Abstract

The present paper reviews the findings of different research studies on the effect of natural ingredients in the Japanese quail (*Coturnix coturnix japonica*) diet on carcass characteristics and meat quality. The results show a relationship between the type and concentration of ingredients used in diets and carcass characteristics and meat quality. The inclusion of medicinal herbs (thyme, black seed, and mint), plants (canola), seeds (chickpea), spices (cinnamon and coriander), worms (earthworms), bee products (propolis), phytochemicals (lycopene), and edible fungi (common mushrooms) in the diet improved carcass quality characteristics compared to the control diets (basal diets). The inclusion of medicinal herbs (spearmint and green tea), spices (cinnamon), vegetables (tomato), plants (verbena and canola), seeds (marijuana), and edible fungi (oyster mushrooms) improved meat quality. In conclusion, the use of ingredients of natural origin in the diet of Japanese quail improves carcass quality characteristics and meat quality.

## INTRODUCTION

The production and consumption of poultry meat in Mexico grew at an average annual rate of 3.2% and 2.7%, respectively, from 2006 to 2015. Poultry consumption amounted to 32.0 kg per capita in 2016 [1]. Domestically, 4,061 M metric tons of poultry were consumed in 2016, and 3,275 M metric tons were produced [2]. The increase in both the production and consumption of poultry can be associated with the greater availability of feed grains, low feed costs, and the accessible price of poultry products as well as the increase in the price of other meats such as pork and beef [1]. To meet domestic demand, in 2016, the import of chicken meat increased to 791 M metric tons [2]. These figures reflect an opportunity to produce more chicken meat locally and nationally, including alternative meats from other species (turkey, duck, guinea fowl, goose, ostrich, pheasant, and quail), without disregarding the criteria of accessibility, price, and quality [3].

The Japanese quail (*Coturnix coturnix japonica*) is native to Europe, northern Africa, and Asia. The raising of Japanese quail has been characterized as economically sustainable and highly productive [4,5], as this quail has a rapid growth cycle (3 to 4 generations per year) and is resistant to diseases [5,6]. With respect to meat production, this bird is considered the smallest of the poultry varieties, making it is easy to manage and to accommodate a large number of birds in a small space. For these reasons, the Japanese quail has also gained importance worldwide for its use in experimental animal models in biological and genetic studies [5]. Therefore, coturniculture, or the raising of quail, is expanding, including the commercialization of different quail products such as fresh or pickled eggs and fresh or frozen carcasses [4,7]. The carcass and meat of Japanese quail are obtained after the quail reaches 35 to 42 d of age and a body weight of 165 to 300 g [5,8]. However, data on quail production are limited regarding the quality of carcass meat destined for processing (31 and 138 M carcasses, for America and Europe, respectively), and the available data are lacking in precision [9].

As is the case for other birds, the integration and management of quail production (breeding, slaughter, processing, and marketing) is essential for maintaining carcass and meat quality [4,5,10]. During the first stage of production, an initiation and growth diet containing a balanced set of nutrients such as carbohydrates, amino acids, essential fatty acids, minerals, vitamins, and water is fed to animals. The quality of quail carcasses is dependent on an adequate and constant supply of energy. The production of birds in temperate climates requires a total of 2,600 to 3,000 kcal/kg of metabolizable energy and, in tropical regions, requires 2,800 kcal/kg [11,12]. Dietary sources commonly used to meet the carbohydrate requirement include grains, such as corn, whereas earthworms, fish, and soybean meal are often used as a protein source [4,8,13, 14]. In addition, soybean and safflower oils can be used as sources of fatty acids [8,14]. To increase the quality of poultry meat, which is mainly affected by oxidative stress, natural ingredients, such as organic compounds (tocopherols), rosemary, green tea, tomato, and honey extracts, have been used in the poultry diet because they contain beneficial compounds with antioxidant activity [15].

Based on the above, the present review summarizes existing research on the inclusion of natural ingredients in the Japanese quail diet to highlight their effect on carcass and meat quality.

## CARCASS AND MEAT COMPOSITION

In relation to chemical composition, the Japanese quail carcass (at 35 to 42 days of age) is composed of 68% water, 19% protein, 10% fat, and 3% minerals [16]. The meat (breasts and legs) is composed of 71% to 74% water, 17% to 23% protein, 2% to 8% fat, and 1.5% to 1.8% minerals [13,16,17]. According to this chemical composition, the protein content of both the carcass and meat is like that found in the literature for the carcass (19.0%) and the legs and breasts (20% and 23%, respectively) of broiler chickens [18,19] but lower than that of red meat (20% to 25%) [20]. In addition, quail meat is considered a valuable source of protein because of its good amino acid profile. Breast and leg meat contain essential amino acids such as cysteine, phenylalanine, isoleucine, leucine, lysine, methionine, tyrosine, threonine, and valine as well as non-essential amino acids such as alanine, arginine, asparagine, glycine, glutamine, histidine, proline, and serine [16]. Different minerals have also been identified in quail meat, including calcium, phosphorus, sodium, potassium, magnesium, iron, copper, and zinc [16,21]. In adequate quantities, these minerals contribute to the formation of the skeletal system and to the health of animals considering that different minerals are involved in metabolic activity and in maintaining the acid-base balance of the body [22].

The fat content of the leg and breast meat of Japanese quails is 3.3% and 2.5% respectively [16]. These values are within the range reported for beef (*Longissimus dorsi*) with light marbling (2.1% to 3.7%) but lower than those reported for lamb meat (*Longissimus dorsi*; 8%) [23,24]. In quail meat, the lipid profile mainly consists of four fatty acids: oleic (C18:1), palmitic (C16:0), linoleic (C18:2), and stearic (C18:0). These four fatty acids represent around 80% of the total fatty acid content in the breast and leg meat of Japanese quail, with C18:1 being present in the highest proportion [16,17,21,25]. With respect to saturated fatty acids (SFAs), C14:0 presents a higher concentration (18.8%) in the leg than in the breast [21]. Meanwhile, with respect to polyunsaturated fatty acids (PUFAs), C18:2 is present the highest concentration, representing 20% of total lipids [16]. On the other hand, the sum of unsaturated fatty acids (UFAs) amounts to 60% in quail meat [16,25]. Additionally, it was reported that birds are able to deposit high amounts of α-linolenic acid in meat when their rations are rich in this fatty acid [16] and when the proportions of UFA:SFA, PUFA:SFA, and PUFAn-6:n-3 in breast meat range from 0.39 to 1.92, 0.20 to 0.73, and 9.31 to 15.3, respectively [16,21,25]. The PUFAn-6:n-3 ratio in meat is considered one of the main evaluation criteria of dietary properties; for broiler meat, a proportion of 16.02 has been reported [26]. The aforementioned ratios of nutritive components are important for maintaining the optimal growth of Japanese quail and for protecting the carcass and meat from oxidative stress [11,25,27]. The Table 1 summarized the chemical composition of quail breast meat in comparison with broiler breast meat.

## EFFECT OF OXIDATIVE STRESS ON CARCASS AND MEAT QUALITY

Oxidative stress occurs as a result of the formation of oxidizing agents, the normal products of aerobic metabolism, or as a result of the intake of oxidizing agents through the diet at a rate that exceeds the capacity of the antioxidant system to eliminate reactive oxygen species, causing significant biological damage in animals and, in particular, affecting the growth of birds [30]. The formation of products from oxidative metabolism can be elevated under stressful conditions and during slaughter, affecting animal welfare and the quality characteristics of carcasses and meat, although suitable breeding practices as well as antemortem conditions can improve the physiological or biochemical state of an animal [30,31].

Regarding the stress experienced by Japanese quails before and during slaughter, the age, sex, and genetics of animals in addition to environmental conditions [8,32–34], feeding, transportation, withdrawal of food, and slaughter method have been found to affect this factor [33,34]. Stress conditions increase the consumption of glycogen and ATP under essentially anaerobic conditions and accelerate the fall of muscle pH [35, 36], resulting in negative changes to carcass characteristics, such as weight loss and reduction in the yield of different cuts of the quail carcass [8,27]. Additionally, in quail meat, these negative changes manifest in the physical properties of meat, including the color and texture [37]; the chemical content in terms of moisture, protein, lipids, and ash [16]; the technological properties, such as decreased water holding capacity, increased weight loss during cooking, and greater drip loss [27,32,37]; and in the biochemical processes with respect to lipid oxidation [27].

To reduce the effects of oxidative stress, antimicrobial compounds (growth-promoter antibiotics, GPAs), including quinoxalines (carbadox and olaquindox), glycopeptides (avoparcin), ionophores (monensin and salomycin), macrolides (tylosin and spiramycin), phosphoglycolipids (flavomycin), streptogramins (virginiamycin), polypeptides (zinc bacitracin), and oligosaccharides (avilamycin), are commonly included in poultry diets for disease control and growth promotion [38]. However, the European Union has banned GPAs, even food-grade antibiotics, because of the possible risk of human pathogenic bacteria developing greater resistance. In other countries in Latin America, efforts are being made to prohibit their use [38–40]. Notably, the growth promotion mechanisms of GPAs are still unknown, yet different hypotheses have been proposed to explain GPAs mechanisms. In particular, GPAs may i) protect nutrients against bacterial destruction, ii) improve nutrient absorption due to the thinning of the small intestine barrier, and iii) reduce the formation of toxins produced by intestinal bacteria [38,41].

In addition, oxidative stress can increase the formation of reactive oxygen species (ROS), including free radicals (hydroxyl, HO^•^; superoxide, O_2_^•^; nitric oxide, NO^•^, alkoxy, RO^•^; and peroxide, ROO^•^), which can react with the proteins and lipids of meat and cause the deterioration of meat quality during storage [42]. Therefore, to retard the oxidative process in meat, synthetic antioxidants such as butylated hydroxyanisole, butylated hydroxytoluene, tert-butylhydroquinone, and propyl gallate are used [42,43]. However, the use of synthetic antioxidants has been associated with potential health risks (e.g., carcinogenesis), which has promoted the establishment of strict regulations in the European Union (Directive of the European Parliament and of the Council No.95/2/EC, dated February 20, 1995) to control their use in foods [43,44].

For this reason, different alternatives are being investigated to improve carcass and meat characteristics, including new nutritional strategies based on the use of natural ingredients (e.g., medicinal herbs, fruits, and plants). Some of the compounds contained in natural ingredients have antimicrobial and antioxidant properties that may exert an effect on animals like those of GPAs and synthetic antioxidants. Their use can possibly reduce stress caused by inadequate management practices during the production and slaughter of birds as well as improve meat quality by increasing oxidative stability through the antioxidant system [6,15,44].

## NATURAL ADDITIVES FOR ENHANCING CARCASS AND MEAT QUALITY

According to the guidelines of the Codex Alimentarius Commission [45], a food additive is “any substance that as such is not normally consumed as a food, nor is it used as a basic ingredient in food, whether or not it has a nutritional value, and whose intentional addition to food for technological purposes (including organoleptic) in its manufacturing, processing, preparation, treatment, packaging, transport, storage or storage phases, is reasonably expected to result (directly or indirectly) by itself or its by-products, in one component of food or an element that affects its characteristics. This definition does not include contaminants or substances added to the food to maintain or improve nutritional qualities.”

On the other hand, the FAO [46] establishes that feed (animal feed) is “all simple or compound material, whether processed, semi-processed or unprocessed, used directly in feeding animals intended for human consumption”. In addition, it defines feed ingredient as a “part or constituent of any combination or mixture that constitutes a feed, whether or not has a nutritional value in animal feed, including feed additives”. Furthermore, ingredients can be “substances of vegetable, animal or aquatic origin, or other organic or inorganic substances”. Meanwhile, a feed additive is considered “any deliberately added ingredient that is not normally consumed as feed, whether it has a nutritional value, and that influences the characteristics of the feed or animal products”.

According to the National Research Council [11], to cover the basal diet requirements of Japanese quail (metabolizable energy = 2,900 kcal/kg), certain nutrients must be included in the diet during the initiation and growth stage, such as i) proteins (24%) and amino acids, specifically arginine (1.25%), glycine+serine (1.25%), histidine (0.36%), isoleucine (0.98%), leucine (1.69%), lysine (1.30%), methionine (0.50%), methionine+cysteine (0.75%), phenylalanine (0.96%), phenylalanine +tyrosine (1.80%), threonine (1.02%), tryptophan (0.22%), and valine (0.95%); ii) fat and macro minerals such as linoleic acid (1.0%), calcium (0.8%), chlorine (0.14%), magnesium (300 mg), non-phytate phosphorus (0.3%), potassium (0.4%), and sodium (0.15%); iii) trace minerals such as copper (5 mg), iodine (0.3 mg), iron (120 mg), manganese (60 mg), selenium (0.2 mg), and zinc (25 mg); and iv) fat-soluble vitamins such as vitamin A (1,650 IU), vitamin D3 (750 ICU), vitamin E (12 IU), and vitamin K (1 mg) and water-soluble vitamins such as vitamin B_12_ (0.003 mg), biotin (2 mg), folic acid (1 mg), niacin (40 mg), pantothenic acid (10 mg), pyridoxine (3 mg), riboflavin (4 mg), and thiamine (2 mg).

Although the abovementioned basic diet is required for the optimal growth of Japanese quail, as confirmed by numerous investigations, it may be possible to increase the yield of carcasses, mainly through reducing the infectious agents that affect the growth and quality of carcasses. Table 2 lists the effects of natural additives in the quail diet on carcasses, as confirmed by research, including the effects of medicinal herbs (thyme, black seed, green tea, duckweed, and mint), plants (verbena and canola), spices (cinnamon), seeds (coriander and chickpea), worms (earthworm), apicultural products (propolis and pollen), chemical compounds (vitamin C, folic acid, and lycopene), and edible fungi (common mushroom).

Table 3 lists the effects of natural additives in the quail diet on meat quality, as confirmed by research, notably the improvement of meat quality through increased oxidative stability. The tested natural ingredients include medicinal herbs (spearmint and green tea), spices (cinnamon and laurel), vegetables (tomato), plants (verbena and canola), seeds (marijuana), insects (black soldier fly), and edible fungi (oyster mushroom).

These additives of natural origin are of interest to the food industry because of their antimicrobial and antioxidant properties, which are commonly associated with the presence of phenolic compounds, such as phenolic acids and flavonoids [42,44].

## ABSORPTION AND METABOLISM OF PHENOLIC COMPOUNDS

Natural ingredients provide a wide range of antimicrobial and antioxidant compounds, such as phenolic compounds, which, in the present case, can modify the intestinal microflora of birds, improving animal health and reinforcing the endogenous antioxidant system responsible for mitigating the effects of free radicals formed during the oxidative stress process [42, 44,65,66]. Phenolic compounds are widely distributed in nature and esterify with glucose or other carbohydrates (glycosides) or are present in the form of free aglycones. The antibacterial and antioxidant properties of these compounds in the bird will depend on their bioaccessibility and bioavailability as well as their absorption under normal physiological conditions following release from the food matrix into the mucosa of the intestine [65,66]. Dietary antioxidants that are non-bioavailable may pass through the intestine without being absorbed and reach the colon. At this point, they may ferment by the action of bacterial enzymes and then contribute toward creating an antioxidant environment by eliminating free radicals, counteracting the pro-oxidant effects of the diet [66,67].

The intake of phenolic compounds in the diet may have adverse metabolic effects associated with the lower efficiency of nutrients, particularly proteins (decreased amino acid digestibility), and with the inhibition of digestive enzymes due to the interaction of their hydroxyl groups with the carbonyl groups of proteins [68,69]. Nevertheless, Brenes et al [70] investigated the effect of including grape seed extract (0.6, 1.8, and 3.6 g/kg) in a diet for broilers during 42 d on productive parameters (weight gain and feed intake), weight of internal organs (pancreas, spleen, and liver), length of digestive organs (duodenum, jejunum, ileum, and caecum), digestibility of phenolic compounds extracted from excreta, and antioxidant activity of the diet and excretions. The results showed that grape seed extract did not affect weight gain (2.2 kg), feed intake (3.9 kg), or the weight of the pancreas, spleen, or liver (0.16%, 0.11%, and 2%, respectively). In the digestive organs, an increase was observed in the length of the duodenum and jejunum (10% and 7%, respectively) in birds fed with 1.8 g/kg of extract compared to the control group. The length of the ileum and caecum also increased (8.3% and 11.9%, respectively) in birds fed with the highest concentration of grape seed extract. Finally, the digestibility of phenolic compounds increased at day 42 between 60% to 69%.

In another study performed by Goñi et al [71], the effect of including 5, 15, and 30 g of grape pomace and 200 mg of α-acetate of tocopherol/kg in a basal diet during 21 d on productive parameters, protein and amino acid digestibility, antioxidant activity of the diet, blood serum, excreta, and lipid oxidation of meat during storage under refrigeration was investigated. The results showed that the inclusion of grape pomace in the diet increased the antioxidant activity of the diets and excreta (33.4% and 15.8%, respectively), although it did not affect antioxidant activity in serum, weight gain (1.84 kg), feed intake (0.86 kg), nutritional efficiency (1.3), or the ileal digestibility of essential and non-essential amino acids. Regarding the consumption and digestibility of phenolic compounds, an increase was observed (37.5% and 56.4%, respectively) in birds supplemented with 30 g of grape pomace/kg compared to the control. In addition, in birds supplemented with grape pomace, the lipid oxidation values of breast and leg meat reduced (42.6% and 30.2%, respectively) at day 7 of refrigerated storage compared to the control. In a research developed by Sohaib et al [72], the influence of dietary quercetin (100, 200, and 300 mg/kg) in combination with α-tocopherol (150, 225, and 300 mg/kg) during 6 wk, on lipid stability of breast meat (male broiler) was evaluated. The results showed that supplementation with 300 mg quercetin+300 mg α-tocopherol/kg exhibited lowest lipid oxidation values (<0.3 mg malondialdehyde/kg) in meat, as well as high antioxidant activity (antiradical DPPH^•^, >50% of inhibition; and ferric reducing antioxidant power, >500 μmol Fe^+2^), which could be associated to the highest values of total phenolic (>100 mg gallic acid equivalent [GAE]/g), quercetin (16.4 mg/kg) and α-tocopherol (38 mg/kg) content.

In another research study conducted by Rupasinghe et al [73], the absorption and distribution of quercetin metabolites in plasma, various tissues, and excreta were evaluated in broilers supplemented with quercetin powder (0, 50, 150, and 300 mg/kg of body weight/d), apple skin extract (50 and 150 mg of total phenol content/kg of body weight/d), and dried apple skin meal (50 mg of total phenol content/kg of body weight/d) for 3 d. Before supplementation, liquid chromatography-tandem mass spectrometry (LC-MS/MS) was performed to identify the phenolic compounds present in the apple powder and apple extract. Quercetin glycosides such as quercetin-3-O-galactoside, quercetin-3-O-glucoside, quercetin-3-O-rhamnoside, quercetin-3-O-rutinoside, and quercetin aglycone were found. The results showed that chickens fed with quercetin powder and apple skin extract at the tested concentrations had quercetin (in glycosylated form more than in aglycone form) in their excreta and plasma as well as in their duodenum and liver; this flavonoid was not detected in the control group. In addition, quercetin was found in chicken meat (breast and leg) supplemented with quercetin powder (300 mg/kg) and apple skin extract (150 and 200 mg/kg) but was not found in the control group. This study shows that quercetin and its glycosides can be absorbed in broilers and, similar to what occurs in humans, are subjected to glucuronidation, sulfation, and methylation once absorbed.

In summary, these results show that phenolic compounds in their glycosylated form, once released from the food matrix, can be absorbed in the intestine by sugar transporters to the enterocyte (through the β-D-glucoside group) and metabolized by broiler chickens, subsequently exerting a pharmacological effect on birds. Meanwhile, phenolic compounds in free form (aglycone) present a low absorption because they lack sugar [74,75].

**Figure 1:**
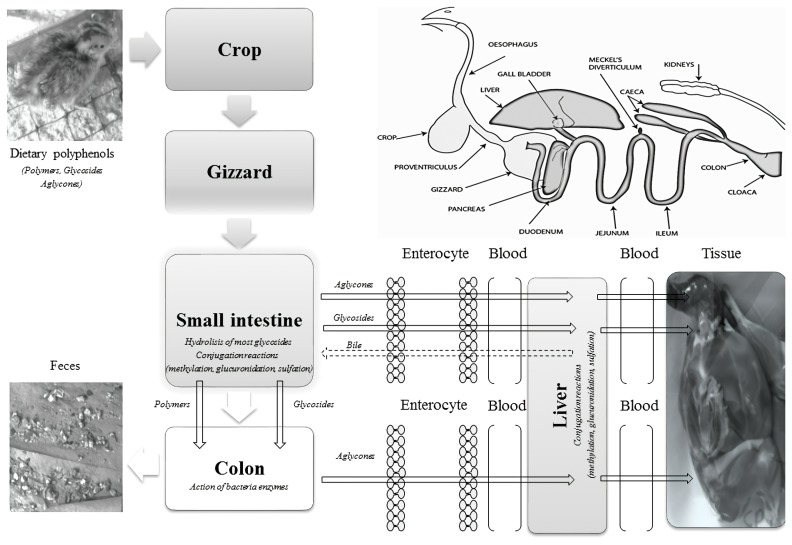
Schematic of dietary polyphenol transport to quail muscle (Addapted from: Ao et al [69]; Brenes et al [79]; Poultry-Hub [80]).

Additionally, Cherian et al [76] evaluated the effect of *Artemisia annua* dried leaves (2% and 4%) on digesta pH and muscle lipid oxidation, and phenolic distribution in dark and white meat of broiler (from day 14 through 42). The results showed that supplementation with *Artemisia annua* reduced pH values of ceca and ileal digesta, and lipid oxidation (thiobarbituric acid reactive substances) in the thigh muscle, which was associated to the increase of phenolic compounds (15.8%) when compared with the control. However, the presence of phenolic compounds in the breast was not affected. In another study, Okarini et al [77] reported the presence of phenolic compounds (68.6, 65.6, and 64.4, respectively) in breast meat of Bali indigenous chicken (20 wk-old), spent laying hen (76 wk-old) and broiler (5 wk-old). Moreover, Vargas-Sánchez et al [78] studied the effect of *Pleurotus ostreatus* powder (1% and 2%) in Japanese quail diet (35 d) to increase the total antioxidant activity of their meat. At day 35, the birds were slaughtering and whole breast removal, and then stored (4°C during 15 d). Each sampling day, an aqueous extract was obtained from the breast an analyzed. The results showed that quails fed with *Pleurotus ostreatus* powder had the highest total phenolic and flavonoid content (>20 mg GAE/g, and >15 mg quercetin equivalents/g, respectively), as well as antiradical activity (DPPH^•^ and ABTS^•+^) when compared with control. The Figure 1 summarizes one of the metabolic absorption mechanisms of polyphenols in the quail diet.

## CONCLUSION

The inclusion of natural ingredients in the diet of Japanese quail such as medicinal herbs, plants, vegetables, spices, seeds, worms, bee products, certain chemical compounds, and edible fungi has the potential to improve carcass and meat quality through reducing oxidative stress. However, this effect depends on the concentration of ingredients and on the type and/or conformation of the compounds present. In addition, these factors can improve or limit the absorption and metabolism of active compounds, enabling or disabling them from acting an antioxidant or antimicrobial agents. Furthermore, high concentrations of certain natural ingredients in the diet can possibly have adverse effects on quail carcasses and meat.

## Figures and Tables

**Table 1:** Chemical composition of quail and chicken breast meat (%)

Items	Quail meat				Chicken meat	
	
	Genchev et al [16]	Hamm and Ang [17]	Cullere et al [21]	Botsoglou et al [25]	Wattanachant et al [28]	Santoso et al [29]
Proximate composition						
Dry matter	26.7	25.4	24.7		25.1	30.1
Protein	21.8	18.9	18.4		20.6	16.3
Fat	2.9	7.9	4.6		0.68	7.7
Ash	1.6	0.9	1.7		1.1	
Essential amino acids						
Cysteine	2.16				0.31	
Phenylalanine	0.97		0.53		3.01	0.6
Isoleucine	1.17		1.09		2.41	0.9
Leucine	2.00		1.77		4.29	1.4
Lysine	2.16		0.46		3.41	1.3
Methionine	0.54		0.49		1.88	0.4
Tyrosine	0.58		1.85		3.03	0.5
Threonine	0.72		1.12		3.02	0.6
Valine	1.22		1.34		2.16	1.0
Non-essential amino acids						
Alanine	1.32		1.89		2.8	1.2
Arginine	1.36		1.10		4.39	1.1
Asparagine	1.99		2.20		3.64	1.7
Glycine	1.07		1.73		2.70	0.5
Glutamine	3.89		3.54		6.35	3.9
Histidine	0.92		0.03		2.9	0.4
Proline	0.99		0.92		1.93	
Serine	0.41		1.18		2.38	0.4
Fatty acids						
Myristic (C14:0)	1.04	0.8	0.25	1.44	0.87	0.4
Palmitic (C16:0)	24.5	20.6	17.8	22.1	31.8	19.9
Palmitoleic (C16:1)	5.69	3.1	1.25	3.32	3.33	4.6
Stearic (C18:0)	8.4	7.1	14.3	12.3	14.6	3.7
Oleic (C18:1)	35.5	44.8	10.2	24.8	37.8	37.2
Linoleic (C18:2)	20	22.9		19.52	7.63	11.7
Linolenic (C18:3)	1.61	0.1		1.21	0.16	0.12
Arachidonic (C20:4)	2.32			3.08	0.45	0.39
∑Saturated fatty acids	33.9		34.6	41.6	48.8	24.2
∑Unsaturated fatty acids	65.9					55.2
∑Monounsaturated fatty acids	41.1			28.6	41.82	
∑Polyunsaturated fatty acids	24.7		42.7	26.26	9.42	
Minerals						
Calcium	0.02	0.02	0.05			0.015
Phosphorus	0.22	0.15	0.89			0.19
Sodium	0.07	0.06	0.25			
Potassium	0.40	0.19	1.5			0.015
Magnesium	0.02	0.03	0.11			
Iron	1.72		<0.001			0.016
Copper	0.37		<0.001			
Zinc	2.02		<0.001			

**Table 2:** Carcass characteristics of Japanese quail supplemented with natural ingredients in their diet

Natural ingredients	Bird management	Relevant results	References
**Medicinal herbs**			
Thyme (*Thymus vulgaris* L.) and black seed (*Nigella sativa* L.)	**Age and weight:** 1 d-old/N.A. **Dose and duration:** 0 and 60 mg of essential oil of TV and NS kg^−1^ of diet and 38 d of supplementation **Husbandry conditions:** 5 birds×cage (50×60 cm), 8 replicates per treatment; temperature (N.A.), moisture (N.A.) and illumination (24 h); vaccine (N.A.) **Slaughter:** fasting time (N.A.) and method (cervical dislocation) **Sex:** not sexed (∅)	At 60 mg TV and NS kg^−1^ (∅); p<0.05 (●) feed intake (662.9 g/bird) (▲) weight gain (2.8%) (●) carcass weight (141.3 g) (●) carcass yield (70.8%) (▼) abdominal fat (22.0%) (▲) liver yield (6.6%) (●) gizzard yield (4.1%) At 60 mg TV kg^−1^ (∅); p<0.05 (▼) feed conversion ratio (5.9%)	Denli et al [47]
Thyme (*Thymus vulgaris* L)	**Age and weight:** 7 d-old/N.A. **Dose and duration:** 0 and 2.5 mL of essential oil kg^−1^ of diet and 150 d of supplementation Husbandry conditions: 18 birds×cage (N.A.), 3 replicates per treatment; temperature (21.7°C), moisture (N.A.) and illumination (24 h during the first wk and 18 h during the next steps of the experiment); vaccine (N.A.) **Slaughter:** fasting time (N.A.) and method (N.A.) **Sex:** female (♀) and male (♂)	At 0 and 2.5 mL kg^−1^ (♀); p<0.05 (●) live weight (172.65 g) (●) carcass weight (106.7 g) (▲) feed intake (1.8%) (●) feed conversion ratio (3.5) At 0 and 2.5 mL kg^−1^ (♂); p<0.05 (●) live weight (150.55 g) (●) carcass weight (99.8 g)	Sengül et al [48]
Duckweed (*Wolffia globosa* L.)	**Age and weight:** 7 d-old/N.A. **Dose and duration:** 0%, 25%, 50%, and 75% powder and 42 d of supplementation Husbandry conditions: 12 birds×cage (N.A.), 6 replicates per treatment; temperature (32°C during 2 wk and 25°C during the next steps of the experiment), moisture (N.A.) and illumination (24 h); vaccine (N.A.) **Slaughter:** fasting time (N.A.) and method (cervical dislocation) **Sex:** not sexed (∅)	At 25% to 75% (∅); p<0.05 (▼) feed intake (9.9%) (●) feed conversion ratio (3.7) (●) carcass yield (76.7%) (●) breast yield (6.8%) (●) leg yield (17.5%) (●) wing yield (20.8%)	Chantiratikul et al [49]
Thyme (*Thymus vulgaris*)	**Age and weight:** 1 d-old/N.A. **Dose and duration:** 0 and 1 g of essential oil kg^−1^ and 35 d of supplementation Husbandry conditions: 15 birds×cage (N.A.), 5 replicates per treatment; temperature (32°C, with a reduction of 3°C per wk), moisture (N.A.) and illumination (24 h); vaccine (N.A.) **Slaughter:** fasting time (N.A.) and method (N.A.) **Sex:** male (♂)	At 1 g kg^−1^ (♂); p<0.05 (●) feed intake (450.5 g/bird) (●) feed conversion ratio (2.8) (▲) live weight (7.1%) (▲) carcass yield (2.5%) (▲) breast yield (6.9%) (●) leg yield (15.4%) (●) abdominal fat yield (1.8%) (●) liver yield (3.3%) (●) heart yield (0.71%) (▼) CFU for E. coli (10.7%) (▲) CFU for *Lactobacillus* (10.4%)	Khaksar et al [50]
Peppermint (*Mentha spicata*)	**Age and weight:** 7 d-old/34.9 g **Dose and duration:** 0%, 1%, 2%, 3%, and 4% of dried leaves and 35 d of supplementation **Husbandry conditions:** 15 birds×cage (N.A.), 4 replicates per treatment; temperature (35°C, with a reduction of 3°C per wk), moisture (N.A.) and illumination (N.A.); vaccine (N.A.) **Slaughter:** fasting time (N.A.) and method (cervical dislocation) **Sex:** male (♂)	At 30 g kg^−1^ (♂); p<0.05 (▼) feed intake (4.7%) (●) feed conversion ratio (3.8) (●) carcass yield (53.5%) (●) breast yield (24.7%) (●) leg yield (17.2%) (▼) CFU for *E. coli* (31.6%) in the ilium	Ghazaghi et al [51]
Peppermint (*Mentha piperita*)	**Age and weight:** 7 d-old/29.6 g **Dose and duration:** 0, 10, 20, 30, and 40 g of dried leaves kg^−1^ and 35 d of supplementation **Husbandry conditions:** 12 birds×cage (N.A.), 5 replicates per treatment; temperature (35°C, with a reduction of 3°C per wk), moisture (60%) and illumination (N.A.); vaccine (N.A.) **Slaughter:** fasting time (N.A.) and method (cervical dislocation) **Sex:** not sexed (∅)	At 20 to 30 g kg^−1^ (∅); p<0.05 (▲) feed intake (3.8%); p = 0.008 (▲) feed conversion ratio (5.3%); p = 0.029 (▼) breast yield (15.1%); p = 0.047 (▼) leg yield (12.5%); p = 0.047 (●) heart yield (0.9%) (●) liver yield (2.5%) (▼) CFU for coliforms (19.7%); p = 0.001 (▲) CFU for lactic acid bacteria (24.3%); p = 0.001	Mehri et al [52]
Green tea (*Camellia sinensis*)	**Age and weight:** 4 d-old/N.A. Dose and duration: 0, 1.25, and 2.5 g of powdered leaves kg^−1^ of diet and 30 d of supplementation **Husbandry conditions:** 17 birds×cage (45×100 cm), 5 replicates per treatment; temperature (21.7°C), moisture (45%) and illumination (N.A.); vaccine (N.A.) **Slaughter:** fasting time (N.A.) and method (N.A.) **Sex:** not sexed (∅)	At 1.25 to 2.5 g kg^−1^ (∅); p>0.05 (●) feed intake (494 g/bird) (●) feed conversion ratio (3.4) (●) carcass yield (66.4%) (●) heart yield (0.87%) (●) liver yield (2.4%) (●) gizzard yield (2.1%)	Kara et al [53]
**Plants**			
Fever tea (*Lippia javanica*)	**Age and weight:** 8 d-old/N.A. **Dose and duration:** 0 and 25 g of powder kg^−1^ and 32 d of supplementation Husbandry conditions: 7 birds×cage (N.A.), 5 replicates per treatment; temperature (N.A.), moisture (N.A.) and illumination (N.A.); vaccine (N.A.) **Slaughter:** fasting time (13 h) and method (cervical dislocation) **Sex:** male (♂)	At 25 g kg^−1^ (♂); p<0.05 (●) feed intake (582 g/bird) (●) feed conversion ratio (N.A.) (●) hot carcass yield (114.3 g) (●) cold carcass yield (76.2 g) (▼) gizzard weight (27.5%) (●) heart weight (1.3 g) (●) liver weight (1.7 g)	Mnisi et al [54]
Canola (*Brassica napus*)	**Age and weight:** 150 d-old/158.3 g Dose and duration: 0%, 2.5%, 5.0%, 12.5%, and 17.5% powder and 150 d of supplementation **Husbandry conditions:** 7 birds×cage (N.A.), 4 replicates per treatment; temperature (N.A.), moisture (N.A.) and illumination (N.A.); vaccines (N.A.) **Slaughter:** fasting time (13 h) and method (stunning with carbon dioxide and subsequent cervical dislocation) **Sex:** female (♀)	At 0 to 17.5% (♀); p<0.05 (●) feed intake (220 g/bird) (●) feed conversion ratio (N.A.) (●) weight gain (71.2 g) (●) gizzard weight (3.1 g) (●) heart weight (1.7 g) (●) liver weight (4.2 g) (●) hot carcass weight (135.2 g) (●) cold carcass weight (133.0 g) (●) wing length (9.4 cm) (●) leg length (3.8 c) At 0 and 5.0% (♀);p<0.05 (▲) small intestine length (6.3%)	Mnisi and Mlambo [55]
**Spices**			
Cinnamon (*Cinnamomum verum*)	**Age and weight:** 420 d-old/N.A. Dose and duration: 0, 100, and 200 mg of oil kg^−1^ and 1 and 2 g of powder kg-1 and 35 d of supplementation **Husbandry conditions:** 15 birds×cage (100×100 cm^2^), 4 replicates per treatment; temperature (37°C to 25°C), moisture (N.A.) and illumination (24 h, fluorescent light of 20-lx intensity); vaccine (N.A.) **Slaughter:** fasting time (12 h) and method (cervical dislocation) **Sex:** male (♂)	At 100 mg kg^−1^ (♂); p<0.05 (▲) live weight (6.2%); p = 0.017 (▼) feed conversion ratio (6.6%); p = 0.003 At 2 g kg^−1^ (♂) (▲) live weight (1.7%); p = 0.017 (▼) feed conversion ratio (1.7%); p = 0.003 At 100 mg kg^−1^ and 2 g kg^−1^ (♂); p<0.05 (●) feed intake (538.4 g/bird); p = 0.802	Mehdipour et al [27]
**Seeds**			
Coriander (*Coriandrum sativum* L.)	**Age and weight:** 3 d-old/N.A. **Dose and duration:** 0.5%, 1%, 2%, and 4% powder and 42 d of supplementation **Husbandry conditions:** 3 birds×cage (19×20×22 cm), replicates per treatment (N.A.); temperature (37°C to 25°C), moisture (N.A.) and illumination (23 h during the first four wk and 14 h during the last two wk); vaccines (N.A.) **Slaughter:** fasting time (N.A.) and method (stunning with carbon dioxide and subsequent cervical dislocation) **Sex:** not sexed (∅)	At 2% (∅); p<0.05 (▲) feed intake (7.6%) (▼) feed conversion ratio (10.1%) (▲) weight gain (6.3%) (▲) carcass yield (4.7%) (▼) abdominal fat (17.6%) (▲) liver yield (17.2%) (●) heart yield (0.78%)	Güler et al [56]
Chickpea (*Cicer arietinum* L.)	**Age and weight:** 1 d-old/9.9 g Dose and duration: 0%, 60% of raw seeds, and 60% of cooked seeds and 35 d of supplementation **Husbandry conditions:** 20 birds×cage (90×90×60 cm), replicates per treatment (N.A.); temperature (35°C to 38°C during the first four days, and a reduction of 5°C per wk during the next steps), moisture (67%) and illumination (N.A.); vaccine (N.A.) **Slaughter:** fasting time (N.A.) and method (cervical dislocation) **Sex:** not sexed (∅)	At 60% raw seeds (∅); p<0.05 (▲) feed intake (2.9%); p<0.01 (●) feed conversion ratio (N.A.) (▲) live weight (7.5%); p<0.01 (▲) carcass yield (5.3%); p<0.01	Obregón et al [14]
**Worms**			
Earthworms (N.A.)	**Age and weight:** 42 d-old/N.A. **Dose and duration:** 0% and 6% powder and 42 d of supplementation Husbandry conditions: 5 birds×cage (N.A.), 5 replicates per treatment; temperature (N.A.), moisture (N.A.) and illumination (N.A.); vaccine (N.A.) **Slaughter:** fasting time (N.A.) and method (N.A.) **Sex:** not sexed (∅)	At 6% (∅); p<0.05 (●) feed intake (N.A.) (●) feed conversion ratio (N.A.) (▲) carcass yield (2.8%) (●) live weight (144.6 g) (●) leg yield (22.3%) (●) breast yield (36.3%) (●) wing yield (9.0%) (●) neck yield (4.1%) (●) chest yield (16.3%) (●) viscera yield (11.9%) (●) bond yield (20.4%) (●) muscle yield (52.7%)	Morón-Fuenmayor et al [13]
Earthworms (*Eisenia foetida*)	**Age and weight:** 1 d-old/7.59 g **Dose and duration:** 0% and 4% powder and 180 d of supplementation **Husbandry conditions:** 3 birds×cage (32×50×30 cm), 6 replicates per treatment; temperature (22.5°C), moisture (64%) and illumination (N.A.); vaccines (N.A.) **Slaughter:** fasting time (N.A.) and method (N.A.) **Sex:** without sexing (∅)	At 4% (∅); p<0.05 (●) feed intake (474.6 g/bird) (●) feed conversion ratio (3.8) (▲) weight gain (8.2%) (▲) carcass yield (4.4%)	Díaz-Cuellar et al [10]
**Bee products**			
Propolis (bee glue)	**Age and weight:** 1 d-old/9.2 g **Dose and duration:** 0, 0.5, 1.0, and 1.5 g kg^−1^ and 35 d of supplementation **Husbandry conditions:** 3 birds×cage (30×50 cm), 10 replicates per treatment; temperature (N.A.), moisture (N.A.) and illumination (24 h); vaccine (N.A.) **Slaughter:** fasting time (N.A.) and method (cervical dislocation) **Sex:** not sexed (∅)	At 0.5 to 1.5 g kg^−1^ (∅); p<0.05 (●) feed intake (625.6 g/bird) (●) feed conversion ratio (N.A.) (▲) weight gain (6.9%); p<0.01 At 1.0 to 1.5 g kg^−1^ (▲) carcass weight (8.2%); p<0.01 At 0 to 1.5 g kg-1; p<0.05 (●) carcass yield (75.4%) (●) abdominal fat weight (1.2 g) (●) liver weight (3.2 g) (●) gizzard weight (3.7 g)	Denli et al [57]
Propolis (bee glue)	**Age and weight:** 8 d-old/9.12 g **Dose and duration:** 0%, 0.5%, and 1% of ethanolic extract and 35 d of supplementation **Husbandry conditions:** 3 birds×cage (50×50×17 cm), 3 replicates per treatment; temperature (N.A.), moisture (N.A.) and illumination (24 h); vaccines (N.A.) **Slaughter:** fasting time (N.A.) and method (N.A.) **Sex:** not sexed (∅)	At 1% (∅); p<0.05 (●) feed intake (615.7 g/bird) (●) feed conversion ratio (2.7) (▲) weight gain (>2%) a 21 d At 0 and 1%; p<0.05 (●) weight gain (224.3 g) (●) live weight (246.3 g) (●) carcass weight (181.7 g) (●) carcass yield (73.7%) (●) liver yield (2.7%) (●) heart yield (1.2%) (●) gizzard yield (3.0%)	Canogullari et al [58]
Pollen	**Age and weight:** 8 d-old/8.29 g **Dose and duration:** 0, 5, 10, and 20 g powder kg^−1^ and 35 d of supplementation **Husbandry conditions:** 3 birds×cage (50×50×17 cm), 3 replicates per treatment; temperature (N.A.), moisture (N.A.) and illumination (24 h); vaccines (N.A.) **Slaughter:** fasting time (N.A.) and method (N.A.) **Sex:** not sexed (∅)	At 5 to 20 g kg^−1^ (∅); p<0.05 (▲) feed intake (6.9%) (●) feed conversion ratio (2.7) (▲) weight gain (>2%) a 28 d At 0 to 20 g kg^−1^; p<0.05 (●) weight gain (223.3 g) (●) live weight (237.5 g) (●) carcass weight (177.3 g) (●) carcass yield (74.6%) (●) liver yield (3.3%) (●) heart yield (1.3%) (●) gizzard yield (3.3%)	
**Chemical compounds**			
Vitamin C and folic acid	**Age and weight:** 10 d-old/46.4 g **Dose and duration:** 0, 250 mg VC, 1 mg FA, and 250 mg VC+1 mg FA (VC+FA) and 42 d of supplementation Husbandry conditions: 10 birds×cage (N.A.), 3 replicates per treatment; temperature (18°C to 22°C), moisture (N.A.) and illumination (N.A.); vaccine (N.A.) **Slaughter:** fasting time (N.A.) and method (N.A.) **Sex:** not sexed (∅)	At VC+FA (∅); p<0.05 (▼) feed intake (1.8%) (●) feed conversion ratio (N.A.) (▼) live weight (4%) (▼) carcass weight (15.3%) (▼) carcass yield (1.3%)	Sahin et al [59]
Lycopene	**Age and weight:** 10 d-old/33.3 g **Dose and duration:** 0, 50, 100, and 200 mg of oil kg^−1^ and 45 d of supplementation **Husbandry conditions:** 3 birds×cage (N.A.), 10 replicates per treatment; temperature (22°C), moisture (57%) and illumination (N.A.); vaccine (N.A.) **Slaughter:** fasting time (24 h) and method (N.A.) **Sex:** not sexed (∅)	At 200 mg kg^−1^ (∅); p<0.05 (●) feed intake (658.3) (●) feed conversion ratio (N.A.) (▲) weight gain (2.0%); p = 0.01 (▲) carcass weight (0.7%); p = 0.01 (▲) carcass yield (0.3%); p = 0.01	Sahin et al [60]
**Mushroom**			
Common mushroom (*Agaricus bisporus*)	**Age and weight:** 7 d-old/N.A. **Dose and duration:** 0%, 0.5%, 1.0%, and 2.0% powder and 35 d of supplementation **Husbandry conditions:** 20 birds×cage (100×100 cm), 3 replicates per treatment; temperature (37°C, with a reduction of 3°C per wk), moisture (N.A.), and illumination (24 h); vaccine (N.A.) **Slaughter:** fasting time (N.A.) and method (cervical dislocation) **Sex:** male (♂)	At 2% (♂); p<0.05 (▲) feed intake (6%); p = 0.005 (▼) feed conversion ratio (6.1%); p = 0.001 (▲) weight gain (12.6%); p<0.001 (▲) liver yield (16.4%); p<0.001 (●) breast yield (27.6%) (●) leg yield (20.5% (●) heart yield (0.84% (●) intestine length (61.2 cm)	Asadi-Dizaji et al [61]

N.A., not available; TV, *Thymus vulgaris* L.; NS, *Nigella sativa* L.; VC, vitamin C; FA, folic acid; (▲), significant increase with respect to the control group; (▼), significant reduction with respect to the control group; (●), without significant differences with respect to the control group; CFU, colony-forming unit.

**Table 3:** Meat quality of Japanese quail supplemented with natural ingredients in their diet

Natural source	Management of birds	Relevant results	References
**Medicinal herbs**			
Green tea (*Camellia sinensis*)	**Age and weight:** 4 d-old/N.A. **Dose and duration:** 0, 1.25, and 2.5 g of powdered leaves kg^−1^ and 30 d of supplementation **Husbandry conditions:** 17 birds×cage (45×100 cm), 5 replicates per treatment; temperature (21.7°C), moisture (45%) and illumination (N.A.); vaccines (N.A) **Slaughter:** fasting time (N.A.) and method (N.A.) **Sex:** not sexed (∅) Meat/temperature and storage time: breast and leg/–20°C until analysis, storage time not specified	At 2.5 g kg^−1^; breast (∅); p = 0.044 (▲) water holding capacity (5.4%) At 2.5 g kg^−1^; leg (∅) (●) water holding capacity (65.8%); p<0.05	Kara et al [53]
Peppermint (*Mentha piperita*)	**Age and weight:** 8 d-old/29.6 g **Dose and duration:** 0, 10, 20, 30, and 40 g of powdered leaves kg^−1^ and 7 to 35 d of supplementation **Husbandry conditions:** 12 birds×cage (N.A.), 5 replicates per treatment; temperature (35°C during the first wk, with a subsequent reduction of 3°C per wk), moisture (60%) and illumination (N.A.); vaccine (NewCastle, only to measure humoral response) **Slaughter:** fasting time (N.A.) and method (cervical dislocation) **Sex:** not sexed (∅) Meat/temperature and storage time: leg/–20°C, 30 d	At 20 to 30 g kg^−1^ (∅) (▼) lipid oxidation-MDA (65.0%); p = 0.001 (▼) cooking loss (14.75%); p<0.001 (▼) drip loss (2.7%); p<0.001 (●) water holding capacity (70.1%) (●) pH value (6.4)	Mehri et al [62]
Peppermint (*Mentha spicata*)	**Age and weight:** 7 d-old/34.9 g **Dose and duration:** 0%, 1%, 2%, 3%, and 4% of powdered leaves and 7 to 35 d of supplementation **Husbandry conditions:** 15 birds×cage (N.A.), 4 replicates per treatment; temperature (35°C during the first wk, with a subsequent reduction of 3°C per wk), moisture (60%) and illumination (N.A.); vaccine (N.A.) **Slaughter:** fasting time (N.A.) and method (cervical dislocation) **Sex:** not sexed (∅) **Meat/temperature and storage time:** breast and leg/–20°C, 90 d	At 1%; breast (∅); p<0.05 (▼) lipid oxidation-MDA (17.5%); p<0.05 At 1%; leg (∅) (▼) lipid oxidation-MDA (16.5%); p<0.05	Ghazaghi et al [51]
**Spices**			
Cinnamon (*Cinnamomum verum*)	**Age and weight:** 420 d-old/N.A. **Dose and duration:** 0, 100, and 200 mg of oil kg^−1^ and 1 and 2 g of powder kg^−1^ and 35 d of supplementation **Husbandry conditions:** 15 birds×cage (100×100 cm^2^), 4 replicates per treatment; temperature (37°C to 25°C), moisture (N.A.) and illumination (24 h, fluorescent light of 20-lx intensity); vaccine (N.A.) **Slaughter:** fasting time (12 h) and method (cervical dislocation) **Sex:** male (♂) **Meat/temperature and storage time:** leg/–23°C until analysis, storage time not specified	At 100 mg kg^−1^ (♂); p<0.05 (▼) lipid oxidation-MDA (17.9%); p = 0.001 (●) pH value (6.2) (▲) water holding capacity (2.4%); p<0.05 (●) cooking loss (22.5%) (●) drip loss (21.4%) At 2 g kg^−1^ (♂); p<0.05 (▼) lipid oxidation-MDA (19.4%); p = 0.001 (●) pH value (6.2) (▲) water holding capacity (3.3%); p<0.05 (●) cooking loss (23.0%) (●) drip loss (22.0%)	Mehdipour et al [27]
Laurel (*Syzygium polyanthum*)	**Age and weight:** 32 d-old/101.94 g **Dose and duration:** 0%, 1%, 2%, 3%, and 4% powdered leaves and 30 d of supplementation **Husbandry conditions:** 5 birds×cage (N.A.), 4 replicates per treatment; temperature (N.A.), moisture (N.A.) and illumination (N.A.); vaccine (N.A.) **Slaughter:** fasting time (N.A.) and method (N.A.) **Sex:** female (♀) **Meat/temperature and storage time:** breast+leg/(N.A.) °C (N.A.)	At 1% to 4% N.A.; p<0.05 (●) fat content reduction (5.5%)	Adriani et al [63]
**Vegetables**			
Tomato (*Lycopersicum esculentum*)	**Age and weight:** 21 d-old/N.A. **Dose and duration:** 0%, 5%, and 10% pulp and 42 d of supplementation **Husbandry conditions:** 6 birds×cage (N.A.), 5 replicates per treatment; temperature (N.A.), moisture (N.A.) and illumination (N.A.); vaccine (N.A.) **Slaughter:** fasting time (N.A.) and method (N.A.) **Sex:** not sexed (∅) **Meat/temperature and storage time:** raw and cooked breast/4°C, 9 d	At 5%; raw and cooked breast (∅); p<0.05 (▼) lipid oxidation-MDA (>10%)	Botsoglou et al [25]
**Plants**			
Fever tea (*Lippia javanica*)	**Age and weight:** 8 d-old/N.A. **Dose and duration:** 0 and 25 g of powder kg^−1^ and 32 d of supplementation **Husbandry conditions:** 7 birds×cage (N.A.), 5 replicates per treatment; temperature (N.A.), moisture (N.A.) and illumination (N.A.); vaccine (N.A.) **Slaughter:** fasting time (13 h) and method (cervical dislocation) **Sex:** male (♂) **Meat/temperature and storage time:** raw and cooked breast/4°C, 24 h postmortem	At 25 g kg^−1^; raw breast (♂); p<0.05 (●) pH value (6.9) (●) L* value (46.3) (●) a* value (3.2) (▲) b* value (53.5) (●) C value (5.8) (●) h value (1.4) (▼) cooking loss (16.5%)	Mnisi et al [54]
Canola (*Brassica napus*)	**Age and weight:** 150 d-old/158.3 g **Dose and duration:** 0%, 2.5%, 5.0%, 12.5%, and 17.5% powder and 150 d of supplementation **Husbandry conditions:** 7 birds×cage (N.A.), 4 replicates per treatment; temperature (N.A.), moisture (N.A.) and illumination (N.A.); vaccine (N.A.) **Slaughter:** fasting time (13 h) and method (stunning with carbon dioxide and subsequent cervical dislocation) **Sex:** female (♀) **Meat/temperature and storage time:** breast/2°C, 24 h post mortem	At 12.5% and 17.5% (♀); p<0.05 (▼) C* value (31.5%) At 0% and 17.5%; (♀) (●) temperature (18.4°C) (●) L* value (49.5) (●) a* value (2.3) (●) b* value (4.7) (●) h* value (1.1)	Mnisi and Mlambo [55]
**Seeds**			
Marijuana (*Cannabis sativa* L.)	**Age and weight:** 7 d-old/N.A. **Dose and duration:** 0, 50, 100, and 200 g kg^−1^ and 42 d of supplementation **Husbandry conditions:** 12 birds×cage (50×90×20 cm), 4 replicates per treatment; temperature (N.A.), moisture (N.A.), and illumination (N.A.); vaccines (N.A.) **Slaughter:** fasting time (N.A.) and method (cervical dislocation) **Sex:** female (♀) and male (♂) **Meat/temperature and storage time:** breast and legs/4°C, 24 h post mortem	At 50 to 200 g kg^−1^; breast (♀) (●) pH values (5.8) (●) weight loss by defrosting (3.8%) (●) L* value (67.3) (●) a* value (14.2) At 50 to 200 g kg^−1^; legs (♀) (●) pH value (6.8) At 200 g kg^−1^; breast (♀); p<0.01 (▼) cooking loss (67.3%) At 50 and 100 g kg^−1^; legs (♀); p<0.01 (▼) L* value (4.9%) (▲) a* value (5.2%) At 50 to 200 g kg^−1^; breast (♂) (●) pH value (5.9) (●) weight loss by defrosting (3.7%) (●) L* value (38.9) At 50 to 200 g kg^−1^; legs (♂) (●) pH value (6.8) At 200 g kg^−1^; breast (♀); p<0.01 (▼) cooking loss weight (48.6%) (▲) a* value (5.5%) At 200 g kg^−1^; legs (♂); p<0.01 (▲) L* value (1.9%) (▲) a* value (11.7%)	Yalcin et al [64]
**Insects**			
Black soldier fly (*Hermetia illucens*)	**Age and weight:** 10 d-old/N.A. **Dose and duration:** 0%, 10%, and 15% larvae and 28 d of supplementation **Husbandry conditions:** 130 birds×cage (N.A.), 5 replicates per treatment; temperature (N.A.), moisture (N.A.), and illumination (N.A.); vaccine (N.A.) **Slaughter:** fasting time (6 h) and method (previous electrical stunning and posterior cervical dislocation) **Sex:** not sexed (∅) **Meat/temperature and storage time:** breast/–40°C, 2 wk	At 0% to 15%; (∅) (●) moisture (75.4%) (●) protein (18.5%) (●) fat (4.6%) (●) ash (1.6%) (●) lipid oxidation-MDA (0.36 mg MDA kg^−1^)	Cullere et al [21]
**Mushroom**			
Oyster mushroom (*Pleurotus ostreatus*)	**Age and weight:** 1 d-old/14.54 g **Dose and duration:** 0, 10, and 10 g of powder kg^−1^ and 35 d of supplementation **Husbandry conditions:** 12 birds×cage (90×90×60 cm), 8 replicates per treatment; temperature (21°C to 36°C), moisture (23.4% to 53.8%), and illumination (24 h); vaccine (without) **Slaughter:** fasting time (3 h) and method (cervical dislocation) **Sex:** male (♂) **Meat/temperature and storage time:** breast/4°C, 15 days	At 0 to 20 g kg^−1^; carcass (♂); p<0.05 (●) carcass weight (133.0 g) (●) carcass yield (61.3%) At 10 and 20 g kg^−1^; breast (♂) (●) moisture (72.7%) (●) protein (22.2%) (●) ash (1.4%) (▼) fat (23.9%); p<0.001 (▼) L* value (8.4%); p = 0.011 (▲) a* value (26.9%); p<0.001 (▼) b* value (12.2%); p<0.001 (▲) water holding capacity (7.2%); p = 0.017 (▼) cooking loss weight (26.8%); p = 0.017 (▼) hardness (30%); p = 0.037 (▼) lipid oxidation-MDA (33.5%); p<0.001	Vargas-Sánchez et al [37]

N.A., not available; (▲), significant increase with respect to the control group (▼), significant reduction with respect to the control group; (●), without significant differences with respect to the control group; MDA, malondialdehyde.
